# Pseudobulbar Affect Mimicking Depression: A Case Report

**DOI:** 10.7759/cureus.26235

**Published:** 2022-06-23

**Authors:** Victor Kekere, Danish Qureshi, Amod Thanju, Patrice Fouron, Tolulope Olupona

**Affiliations:** 1 Psychiatry, Interfaith Medical Center, Brooklyn, USA

**Keywords:** psychiatric comorbidities, antidepressant, nuedexta, depression, pseudobulbar affect

## Abstract

Pseudobulbar affect (PBA) is a neurological condition that is associated with short periods of involuntary, sudden, and inappropriate emotions such as crying or laughing, which are mood incongruent. An accurate estimate of the prevalence of PBA is hard to obtain due to varying diagnostic criteria and variable patient populations. The cause of PBA is not known, but current evidence suggests dual etiology. A neural circuit dysfunction and an abnormality of neurotransmitters that regulate motor expression of emotions. PBA can easily be mistaken for a depressive disorder due to the overlap of symptoms. Moreover, patients with PBA may have a major depressive disorder (MDD) or other depressive disorders. Therefore, it is essential to recognize and treat PBA as well as possible psychiatric comorbidities. We present a case report of a 59-year-old man with no past psychiatric history who presents with paroxysms of episodes of crying for the past one year. He endorsed feelings of hopelessness and poor concentration. MRI of the brain revealed bilateral basal ganglia and a thalamic infarct. The patient was treated with citalopram. This case describes a unique presentation of pseudobulbar affect mimicking depression.

## Introduction

Pseudobulbar affect (PBA) is a neurological condition that is associated with short periods of involuntary, sudden, and inappropriate emotions such as crying or laughing, which are mood incongruent [1]. Many are embarrassed by this inappropriate emotional display, which they cannot voluntarily prevent from happening or stop when it has occurred [2]. Thus, there is a dissociation of emotional expression from emotional experience [2]. Clinical syndromes of abnormal emotional expression in patients with brain lesions have been described since the 19th century with various nomenclatures, including pathological laughter and crying, involuntary emotional disorder, emotional lability, emotional incontinence, and pseudobulbar affect [2-4]. Historically, PBA has been misdiagnosed, underdiagnosed, and undertreated [1,3]. An accurate estimate of the prevalence of PBA is hard to obtain due to varying diagnostic criteria and variable patient populations. It is estimated that up to two million Americans have PBA [5]. Research suggests that the range of estimates of the prevalence of PBA in various neurological disorders is high, ranging from 5% to well over 50%. It is especially common in patients with amyotrophic lateral sclerosis, multiple sclerosis, traumatic brain injury, Alzheimer’s disease, stroke, extrapyramidal and cerebellar disorders, and brain tumors. Diagnosable psychiatric disorders have been reported to be higher in patients with PBA. Studies have also shown that about 30%-35% of patients with PBA are depressed [6]. PBA is therefore commonly misdiagnosed as a mood disorder, particularly depression or bipolar disorder. This case reports a unique presentation of pseudobulbar affect mimicking depression.

## Case presentation

**Figure 1:**
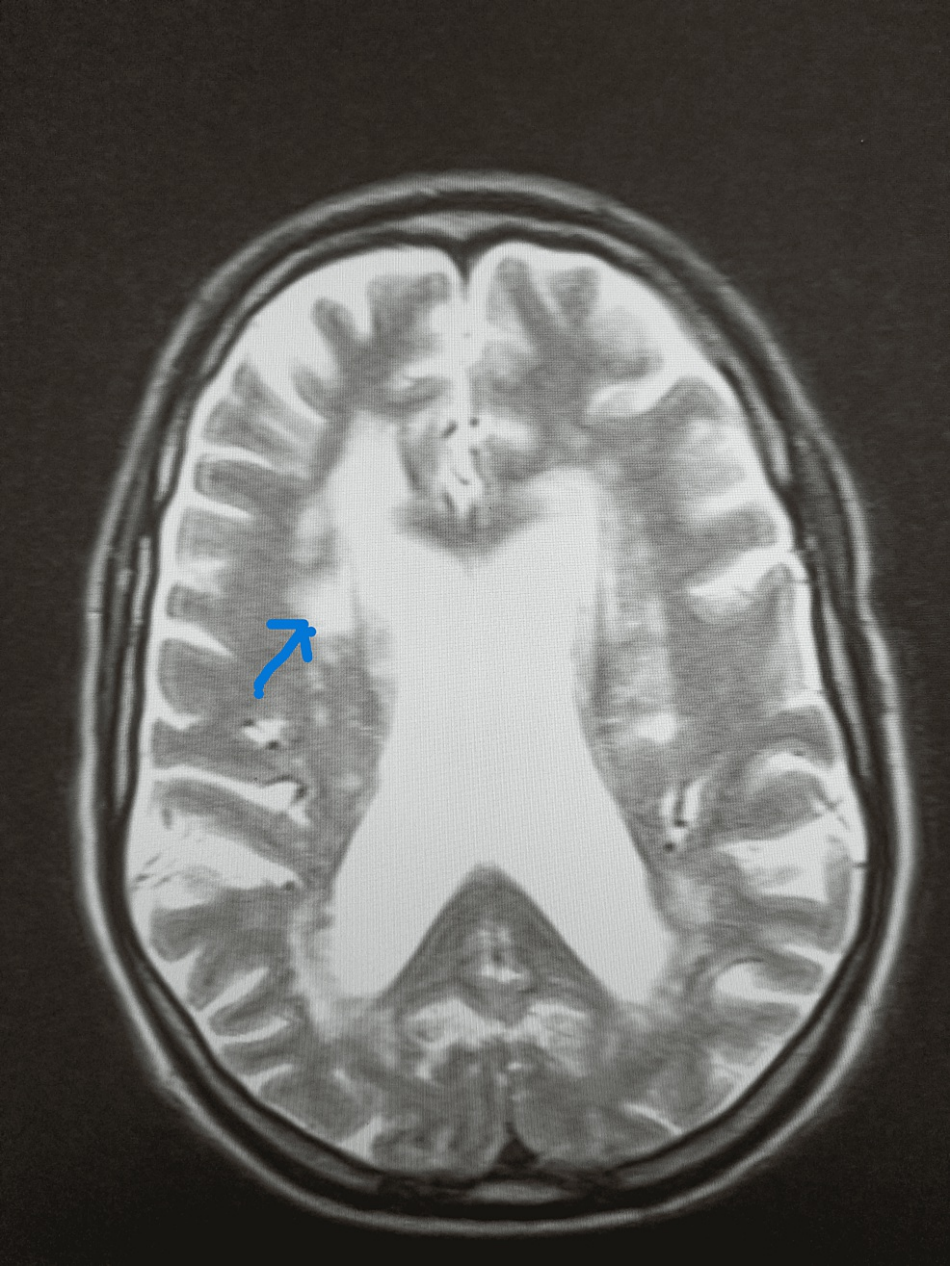
MRI showing nonspecific periventricular and deep white matter degenerative changes.

**Figure 2:**
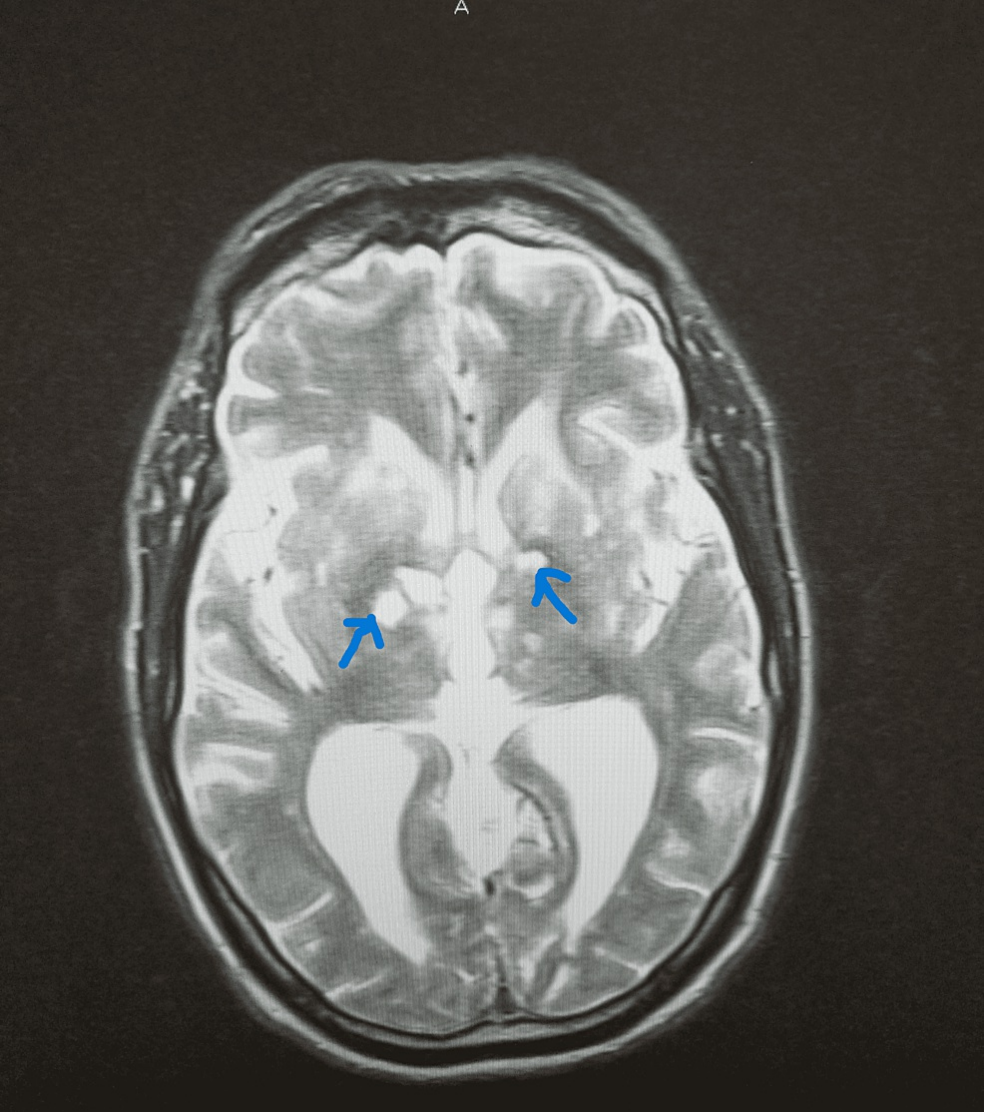
MRI brain w/stem without contrast showing bilateral thalamic and basal ganglia infarcts.

We present the case of a 59-year-old male with no past psychiatric history and a past medical history of hypertension and polysubstance abuse who was admitted to the medical unit for complaints of chest pain. Acute coronary syndrome (ACS) was ruled out following low troponin levels and normal sinus rhythm on EKG. The patient was managed by the medical team for syncope and altered mental status in a patient with mild cognitive impairment likely due to organic brain syndrome. A psychiatric consultation was obtained for depression following the patient's wife's report of paroxysm of episodes of crying for hours over the past one year. The patient endorsed poor concentration and feelings of hopelessness. However, he denied depressed mood, anhedonia, excessive guilt, fatigue, or suicidal ideation, intent, or plans. He denied changes in her sleep, appetite, or recent weight gain or loss. The patient was observed with episodes of crying that were sudden and involuntary. He reported feeling embarrassed by the crying episodes. Center for Neurologic Study-Liability Scale (CNS-LS) for pseudobulbar affect was administered to the patient with a score of 22. A CNS-LS score of 13 or more is suggestive of PBA. The CT scan of the head without contrast shows nonspecific ventricular hypo-densities likely related to microvascular ischemia, and the MRI of the brain was significant for bilateral chronic basal ganglia and thalamic infarcts as well as nonspecific periventricular and deep white matter degenerative changes (Figures 1, 2). The team recommended the patient be treated with citalopram. The patient was discharged after five days with some improvement and referred to the outpatient clinic. He was lost to follow-up. 

## Discussion

Although the cause of PBA is unknown, current evidence suggests the involvement of both neural circuit dysfunction and abnormalities of neurotransmitters that regulate motor expression of emotions [1]. Pseudobulbar affect results from the disruption of the cerebro-ponto-cerebellar circuit, which decreases the threshold of the expression of emotions. This neural tract controls the limbic and motor descending pathways of the brainstem and cerebellum. The cortical inputs to these circuits are normally responsible for the inhibition of inappropriate affect [1]. Existing evidence also proposes that abnormal dopaminergic, glutaminergic, and serotoninergic neurotransmission may play a part in the development of pseudobulbar affect. Imaging studies have shown a reduction in dopamine and serotonin neurotransmission and an increase in glutamate neurotransmission. Therefore, treatment entails increasing dopamine and serotonin neurotransmission and reducing glutamate neurotransmission [7].

A very difficult aspect of pseudobulbar affect is distinguishing it clinically from depressive disorders. Clinicians can easily misdiagnose the exaggerated or disproportionate crying seen in pseudobulbar affect for depression [4]. Table 1 highlights the difference between PBA and depression. In our case presentation, the patient had some symptoms of depression including, paroxysm of crying episodes, hopelessness, and poor concentration, but did not meet the Diagnostic and Statistical Manual of Mental Disorders, Fifth Edition (DSM-5) criteria for major depressive disorder (MDD) or other depressive disorders. Given the high prevalence of depression in patients with PBA, it is important to diagnose any depressive disorder and treat it appropriately. A case report by Espiridion et al. showed the possibility of having a major depressive disorder in a patient with pseudobulbar affect and the importance of treating both. The patient had a similar presentation as our patient with pseudobulbar affect but had other symptoms of depression and met the DSM-5 criteria for a major depressive disorder. The patient was treated for major depressive disorder with mirtazapine and pseudobulbar affect with Nuedexta with significant improvement in both symptoms [8]. Additionally, early identification and treatment of PBA are essential regardless of the cause. PBA may be objectively measured by validated scales like the Center for Neurologic Study-Liability Scale (CNS-LS), which comprises seven questions, with each question getting a score from 1 to 5, with a total of 35 points and the Pathological Laughing and Crying Scale (PLCS), which has 18 questions with a score range of 0-3 for each question. A score of 13 or more on the CNS-LS is suggestive of PBA with high sensitivity and specificity [4,5,9,10]. The use of the DSM-5, the performance of a structured interview, and the use of validated scales may minimize confusion between PBA and depression.

**Table 1:** Difference between pseudobulbar affect and depression. PLCS: pathological laughing and crying scale, PHQ-9: patient health questionnaire-9, DSM-5: Diagnostic and Statistical Manual of Mental Disorders, Fifth Edition, FDA: Food and Drug Administration, CNS-LS: Center for Neurologic Study-Liability Scale, SSRI: selective serotonin reductase inhibitor, and SNRI: serotonin-noradrenaline reuptake inhibitor.

Measures	Depression	Pseudobulbar affect
Nature of crying	Mostly controllable, stops when mood changes	Uncontrollable
Nature of crying	Onset and duration are defined by the mood	Unpredictable, sudden onset, and brief episodes
Nature of crying	Congruent with mood	Usually incongruent with mood
Nature of laughter	Usually not present	Inappropriate, sudden onset, and incongruent with mood
Screening	PHQ-9, Hamilton depression rating scale	CNS-LS, PLCS
Etiology	Multifactorial	Usually secondary to a neurologic condition
Diagnosis	History and DSM-5	History and imaging
FDA approved treatment	SSRI, SNRI, psychotherapy	Nuedexta
Most common psychiatric comorbidity	Anxiety	Depression

The aim of treatment of PBA is to decrease the severity and frequency of episodes with the goal of improving well-being. The main targets of pharmacotherapy are norepinephrine, serotonin, or glutamate, using tricyclic antidepressants (TCAs), selective serotonin reductase inhibitors (SSRIs), and, most recently, the cough suppressant dextromethorphan [6]. Dextromethorphan inhibits glutamatergic transmission by acting as a noncompetitive N-methyl-D-aspartate receptor (NMDA) receptor antagonist and sigma 1 (σ-1) receptor agonist [6]. The first treatment that was FDA-approved for PBA was Nuedexta in 2010. Nuedexta is a fixed combination dosage capsule with 20 mg of dextromethorphan hydrobromide and 10 mg of quinidine sulfate. Monotherapy with dextromethorphan has not been as effective because it is rapidly metabolized by CYP2D6 to dextrorphan, which is not able to cross the blood-brain barrier [1,6]. Quinidine sulfate blocks the hepatic metabolism of dextromethorphan by inhibiting CYP2D6, which slows down the metabolism of dextromethorphan and thus increases brain exposure [1,6]. Nuedexta was not used in this patient due to cost and lack of access. The cost of Nuedexta is significantly more expensive than generic dextromethorphan, quinidine, or other off-label use SSRIs [11].

## Conclusions

PBA is a neurological disorder characterized by a lack of voluntary control over affective expression; it is a disorder of disinhibition. PBA is often unrecognized and misdiagnosed. Raising awareness of PBA through the education of physicians, ancillary healthcare providers, patients, families, caregivers, and the public will permit an increase in recognition of PBA. Although SSRI and TCA can be used for both depression and PBA, dextromethorphan hydrobromide and quinine sulfate (Nuedexta) are the only FDA-approved medications for PBA with clinical efficacy. The recognition and diagnosis of PBA as well as education of the patient and caregivers about the illness are essential for appropriate pharmacotherapy and improved quality of life.
